# Virtual Reality and Simulation Videos as Effective Training Tools for Creating Safe and Inclusive Environments for Transgender People

**DOI:** 10.3390/nursrep14010004

**Published:** 2023-12-28

**Authors:** Jesús Manuel García-Acosta, Francisco Javier Castro-Molina, Naira Delgado, Olga Díez-Fernández, Natalia Rodríguez-Novo, María Elisa de Castro-Peraza, Nieves Doria Lorenzo-Rocha, Jesús Miguel Torres-Jorge, Alfredo David Fernández-Martínez, María Andreína Castellano-Fuenmayor

**Affiliations:** 1 The Canary Islands Public Health Service, Tenerife, 38071 Canary Islands, Spain; extjgarciaa@ull.edu.es (J.M.G.-A.); mcastrop@ull.edu.es (M.E.d.C.-P.); nlorenzr@ull.edu.es (N.D.L.-R.); alu0101098906@ull.edu.es (M.A.C.-F.); 2 Nuestra Señora de la Candelaria School of Nursing, University of La Laguna, Tenerife, 38010 Canary Islands, Spain; 3 Department of Cognitive, Social, and Organisational Psychology, University of La Laguna, Tenerife, 38200 Canary Islands, Spain; ndelgado@ull.edu.es; 4 Department of Education, Vocational Training, Physical Activity and Sport, Regional Government of the Canary Islands, Tenerife, 38071 Canary Islands, Spainafermar1@gobiernodecanarias.org (A.D.F.-M.); 5 Department of Nursing, University of La Laguna, Tenerife, 38200 Canary Islands, Spain; nrodrigu@ull.edu.es; 6 Department of Computer and Systems Engineering, University of La Laguna, Tenerife, 38200 Canary Islands, Spain; jmtorres@ull.edu.es

**Keywords:** virtual reality, instructional film and video, students, nursing, education, nursing, nursing education research, diversity, equity, inclusion, transgender persons

## Abstract

Background: University education is undergoing a paradigm shift towards active methodologies, such as virtual reality and training videos, which have proven to be valuable resources, especially in the health sciences. The scarcity of existing research on the topic prompted us to conduct this study, which seeks to measure the knowledge gained from the aforementioned tools by users, their level of satisfaction with them, and their perceived utility. Methods: This is a quasi-experimental intervention study analysing the impact of virtual objects as learning resources for undergraduate nursing students. Results: Fifty-four participants completed the training, yielding highly significant differences between their mean scores, with a high statistical power and a large effect size. A total of 85.46% of participants confirmed that the virtual resources helped them considerably to empathise with the experiences of trans people in healthcare settings. Students were comfortable using the virtual resources, very satisfied with the methodology employed, and would recommend the training received. Conclusions: University teaching must adapt to meet the current legislations and changing health needs of society, and teaching staff must be prepared to implement new active teaching methodologies that make learning a more dynamic process. Considering these results, our study serves as a guide for other nursing educators who seek to promote inclusive healthcare regarding gender diversity. This study is not registered.

## 1. Introduction

The World Professional Association for Transgender Health (WPATH) is a non-profit, interdisciplinary, professional, and educational organisation whose members promote high-quality care for transgender and gender non-conforming people internationally. It regularly publishes the Standards of Care (SOC), an international reference manual for the specific health and care needs of transgender and gender non-conforming people. As indicated in this manual, the generic term trans should be used to describe people whose gender identity and/or gender expression does not correspond to the sex assigned at birth [1].

Following this approach, the term trans is commonly used in the scientific literature to refer to people whose gender identity or gender expression differs from internationally accepted social roles in the context of cisheteronormativity [2].

As the existing scientific evidence demonstrates, the vast majority of nurses feel unprepared and lack the necessary experience, skills, and knowledge to provide care tailored to the specific needs of trans people [3,4,5], which can present a number of barriers to healthcare [2,6].

According to the Code of Ethics for Nurses published by the International Council of Nurses (ICN), nurses should promote an appropriate environment in which human rights are respected, maintain their competence through continuing education, and actively contribute to knowledge acquisition based on the best available scientific evidence and the latest research [7].

In particular, in Spain, the National Agency for Quality Assessment and Accreditation (NAQAA) is responsible for publishing the white book of the nursing degree, where the competences and learning outcomes of each subject are set out. This publication dates from 2004, does not include training for gender diversity, and is not adjusted to the health needs of the current population [8].

On this basis, the current curricula for nursing degrees in Spain must be reviewed as they do not provide formal training on gender diversity in general or on transgenderism in particular [5,9]. This is in spite of the fact that the recently approved legislation recognises the right of trans people to be cared for by health professionals who are familiar with their reality and encourages training and research on this topic at universities [10]. This creates an important opportunity in university education.

Clinical competence is necessary for all health professionals [11], just as clinical decision making is considered an essential cognitive skill for nurses. Clinical decisions are a systematic, critical thinking process that nurses engage in on an ongoing basis, whereby they identify and manage the health needs of users as they arise [12]. It is against this backdrop that an opportunity arises to implement trans-competent training tailored to the emerging health needs of the population at present.

The European Higher Education Area (EHEA) has created new challenges and requirements in knowledge management, a field in which the current educational paradigm is shifting towards a student-centred educational model (SCEM) [13]. This constructivist model promotes more reflective, responsible, and autonomous learning, resulting in the development of professional skills in a more efficient manner [14]. It is essential for nursing teachers to adopt new teaching strategies that spark students’ interests while promoting skill and knowledge acquisition [15]. As such, the use of Information and Communication Technologies (ICTs) offers a helpful alternative to conventional teaching models [16,17].

ICTs include procedures and techniques used to store and convey information based on IT, telematics, and multimedia [16]. The use of ICTs is increasing exponentially in health sciences due to the extensive use of mobile devices [16]. Compared to traditional education methods, the use of ICTs makes a greater contribution to improving students’ self-confidence, increasing their level of knowledge, and enhancing skills acquisition [17,18,19].

The most common forms of ICT in education are virtual learning objects (VLOs) and virtual learning environments (VLEs) [16,20]. Of these, one of the best-known forms of simulation, which is also gaining traction in the health sciences, is virtual reality (VR), in which experiences are fully immersive thanks to stereoscopic head-mounted displays [12,21,22,23]. The popularity of VR stems from its positive impact on learning outcomes and development of critical thinking, decision making, and clinical reasoning and judgement [12]. Moreover, under these conditions, students can experiment and learn to master new skills in simulated environments that are very similar to real life without endangering themselves or users [18,19]. Therefore, VR can help learners develop communication skills [11] and non-technical skills, such as awareness and understanding, which are essential for empathy [24], as well as stress management, leadership, and teamwork [12].

Simulation or training videos (SVs) as VLOs are a common alternative within the context of ICTs for higher education and health sciences. Simulation has emerged as a teaching process and an active learning method in which real-life situations similar to those that students may face in their healthcare practice are recreated [13,25]. In fact, simulation combines the complexity of both theoretical and practical learning [13], making it one of the active methodologies with the greatest benefits for student training [26].

In the literature, SVs are regarded as a valuable, innovative, and effective learning strategy [27], as well as a widely used resource for the acquisition of communication skills, such as motivational interviewing [27,28]. Most of the available evidence groups simulation results into three categories: satisfaction, degree of knowledge acquired/learning, and behaviour [29].

When exploring nursing students’ perceptions, the vast majority state that these teaching options are beneficial for the acquisition of knowledge and the development of decision-making skills [12]. They also report high levels of satisfaction and interest when using them, greater self-confidence, and higher self-perceived competence [11]. In general, students view the use of VR as a safe, appealing, and beneficial methodology [23].

Constructivist models generate the opportunity for students to be able to analyse and reflect on the professional practice proposed to them, reflect on it, and help them to relearn and change their ways of acting [30].

Therefore, under a constructivist approach of reflective and autonomous learning, this study proposes a hybrid learning model based on two virtual methodologies: training videos and virtual reality. Thus, the main objective of this study is to evaluate the effectiveness of these teaching methodologies to train nursing students in the acquisition of knowledge on the generation of safe and inclusive environments for trans people.

## 2. Materials and Methods

### 2.1. Objectives

The main objective was to analyse the impact of an educational intervention using virtual objects (VR and SVs) as active learning methodologies.

The specific objectives were as follows:

1. To create and validate the educational audiovisual resources to be used.
2. To assess their impacts by:
  - Measuring knowledge growth.
  - Analyse the changes in content, attitudes, and procedures.
  - Measure the degree of satisfaction with the methodology used.

### 2.2. Participants

The study participants were recruited among fourth-year undergraduate nursing students (n = 60) in the 2022–2023 academic year at the Nuestra Señora de Candelaria School of Nursing (EUENSC), affiliated with the University of La Laguna, on the island of Tenerife (Spain). Most of the researchers who were involved in implementing the tools and creating and validating the resources (VR and SVs) were teachers at EUENSC.

The inclusion criteria were fourth-year nursing students enrolled in the practicum module, who had signed the consent form.

A non-probability purposive sampling method was used. Participation was voluntary and did not involve any academic evaluation or financial compensation for any of the participants.

Students who refused to participate or did not complete 100% of the training were excluded.

### 2.3. Design and Methodology

This was a quasi-experimental intervention study analysing the impact of an educational intervention using virtual learning resources as teaching tools. This design was chosen because there was no control group.

The study was carried out in two phases. In the first phase, virtual resources were produced by simulating three different potential scenarios that students could face in their future professional careers: a mental health consultation, a paediatrics consultation, and a primary care consultation. In these scenarios, the user or patient was invariably a trans person. Two versions of each scenario were recorded: one exclusionary and one inclusive.

**Figure 1:**
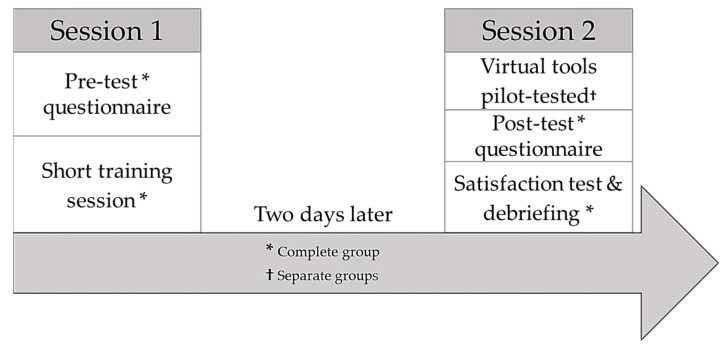
Intervention planning.

In the second phase, the educational intervention was implemented using the tools created. The intervention was carried out in two sessions (Figure 1). In the first session, a pre-test on knowledge was administered and then a short training session was delivered in the form of a lecture. Two days later, in the second session, the virtual tools were pilot tested: two in an SV format (the adult and mental health consultations) and another in a VR format (the paediatrics consultation).

In the form of a lecture, aspects, such as basic concepts related to sexual identity and orientation, the difference between sex and gender and good practices (respecting identity, name, and pronoun meaning), and cares for specific needs, such as hormonal, surgical, pregnancy, and perinatal care, were addressed.

In the realisation of virtual scenarios, theoretical aspects were addressed, as well as non-judgmental aspects to foster empathy and respect, clearly differentiating between exclusionary and inclusive environments. For example, in the paediatrics consultation, the actress played a 14-year-old trans girl. In the exclusionary scenario, the nurse continually addressed her with the male name (deadname) and the male pronoun, assuming that she was a homosexual boy. Whereas in the inclusive scenario, the meaningful name was respected at all times and the feminine pronoun was used, without prejudging her sexual orientation.

**Figure 2:**
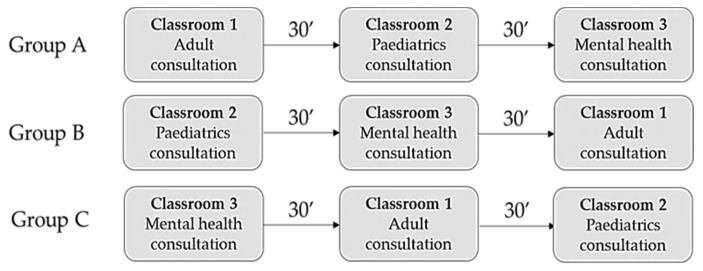
Virtual tools pilot-test flowchart.

Prior to the intervention, students were randomly divided into three groups. As they entered the classroom, they were assigned an identification number (ID). Group A consisted of participants with IDs from 1 to 20, group B with IDs from 20 to 40, and group C with IDs > 40. This allowed all groups to work on different scenarios at the same time. Each scenario lasted 30 min, and once the 2 versions had been viewed, a small group discussion or debriefing was held. Figure 2 shows the flowchart for this process.

Once they completed the three scenarios, the three groups were brought together in a classroom where the post-test questionnaire and satisfaction test were administered, followed by a debriefing/discussion in large groups made up of representatives from the three groups.

### 2.4. Instruments

As a measuring instrument, an ad hoc questionnaire consisting of 15 questions (Appendix A, underlining indicates the correct answer.) was developed and used as a pre- and post-test to measure the increase in knowledge through skills development in terms of: (1) content: current state of the pathologisation of transgenderism, the concept of transgender, the difference between sex and gender, barriers to healthcare and knowledge about healthcare standards specific needs of trans people (questions 1–5); (2) attitudes: resources to express sensitivity and respect for trans people and where these are collected (questions 6–10); and (3) procedure: creating inclusive and safe healthcare environments and examples of practices for their implementation (questions 11–15).

To validate the 15 questions, the questionnaire was submitted to a nominal and multidisciplinary group of 13 experts (of which 8 had a PhD) who had to reach a consensus on the face validity and understandability of the questions. The expert characteristics were as follows: more than five years’ experience in their field, with at least two publications on transgenderism in journals indexed in JCR or SJR, and with proven knowledge and experience of teaching, research, and IT.

To assess utility and satisfaction with the methodologies, a self-administered questionnaire was prepared using Google Forms^®^, limiting the number of responses to one per IP (Internet Protocol) address. The questionnaire contained a total of 15 items, 3 of which covered sociodemographic variables. The other items were Likert-style questions.

The online tool Mentimeter^®^ was used as an ICT resource for the final briefing once the training was completed and the students were asked to list three words describing what they understood to be a safe and inclusive environment for trans people.

### 2.5. Data Processing and Analysis

A statistical analysis was performed using the advanced data analysis software SPSS Statistics^®^ (version 29.0) from IBM (IBM Corp. Released 2019. IBM SPSS Statistics for Windows, Version 29.0. Armonk, NY, USA). This programme offered a wide range of functions for data analysis, including descriptive and inferential statistics. This allowed us to analyse the study data comprehensively and thus draw sound conclusions.

A descriptive analysis was conducted by calculating measures of central tendency and dispersion for quantitative variables, as well as distribution measures for qualitative variables. All calculations were performed with a 95% confidence level and an error of 5%. The independent variable was the implementation of the educational tool using virtual resources.

### 2.6. Ethics and Confidentiality

This study was part of the research project ENF21/09: ‘TranSkin: generating inclusive healthcare environments centred on trans* people’, approved in a competitive regional call funded by the Canary Islands Foundation for Health Research (FIISC) and promoted by the Regional Ministry of Health of the Government of the Canary Islands and the Canary Islands Health Service.

The study adhered to the guidelines set out in the Declaration of Helsinki and the laws and regulations in force in Europe and Spain to ensure the protection of the rights and safety of research participants, to promote the quality of research and to protect the reputation of science.

The researchers informed the participants about the study objectives and procedures and requested that they sign the informed consent form. Once the forms were signed and returned, the researchers proceeded to examine the subjects and collect the data required for the research.

The processing, communication, and transfer of personal data from all participating subjects complied with the provisions of the Spanish Organic Law 3/2018 of 5 December on Personal Data Protection and Guarantee of Digital Rights [31] and the General Regulation (EU) 2016/679 of the European Parliament and of the Council of 27 April 2016 on the protection of natural persons with regard to the processing of personal data and on the free movement of such data (GDPR) [32].

To ensure the confidentiality of the data collected from the study participants, the data were attributed unique code numbers that prevented the participants from being identified. Moreover, access to the data was restricted to the principal investigator, his team of collaborators, the sponsor’s representative in charge of monitoring, the auditor in the event of an audit, the Ethics Committee for Medical Research (ECMR), and the Spanish Health Authorities.

The ECMR approved the conduct of this study at the EUENSC under registration code: CHUNSC_2021_55.

## 3. Results

The results of this study show that VLOs in both VR and SVs formats have a significant impact on the learning of 4th-year nursing students. These students significantly improved their knowledge and skills after training. In addition, both methodologies were well received by the students showing a high degree of satisfaction.

Out of a total of 60 students invited to participate, 56 signed the consent form and 54 of these completed the training. A total of 83.40% were women aged between 20 and 25 years old. The descriptive pre- and post-test data are shown in Table 1.

**Figure 3:**
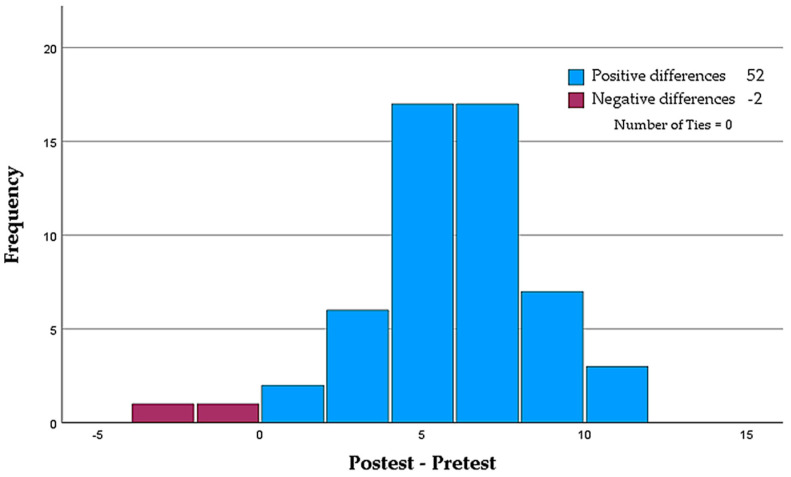
Paired-samples Wilcoxon signed-rank test.

Statistically significant differences were found between the mean scores obtained using the Wilcoxon non-parametric test for paired samples (Z = −6.304; sig. = <0.001; 95% CI). As shown in Figure 3, 52 of the 54 participants experienced an increase in their level of knowledge.

The statistical power and effect size were calculated and found to be 1 and 2.69 (Hedges’ correction), respectively. Therefore, it is safe to conclude that our data are highly extrapolable to the population and that all the variances found in our sample can be explained by the independent variable.

An analysis by skill blocs revealed skills development in all three blocs, with a clear increase in knowledge (in terms of content: specific needs of trans people; attitudes: sensitivity and respect towards trans people; and procedure: creation of inclusive and safe healthcare environments).

McNemar’s test showed no significant differences in questions 6, 7, and 9 of the attitudinal skills bloc (Appendix A) when analysed pairwise and dichotomously (correct/incorrect) (Table 2).

This result is explained by the fact that the number of correct and incorrect responses in the pre- and post-tests is the same, with no differences found. For the other 12 questions, statistically significant differences were found.

In terms of the student satisfaction with and perceived utility of the virtual resources that were pilot tested, 78.18% of them were very likely to recommend the training for other health sciences disciplines; 74.55% were very satisfied with the knowledge acquired; and 90.91% felt that their level of knowledge about trans issues increased greatly or considerably. When asked about satisfaction with the methodologies, more than 67.00% were very satisfied and more than 86.00% reported feeling comfortable or very comfortable with VR.

Regarding the organisation and delivery of the workshop, 76.36% stated that the workshop was very well structured, 89.09% rated the teacher as very good, and 87.27% reported that the teacher had a good command of the subject matter and was keen for the students to understand the contents of the workshop.

One important finding was that 85.46% of the participants confirmed that the virtual simulations provided through SVs and VR helped them greatly or considerably to empathise with the experiences of trans people in healthcare settings.

**Figure 4:**
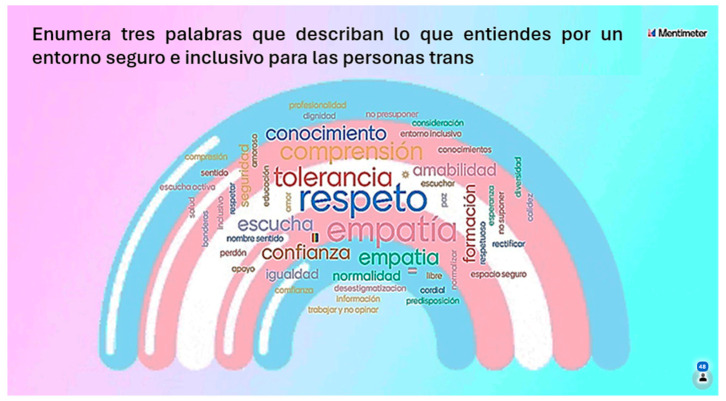
Graphical representation of student response with Mentimeter^®^.

Finally, the Mentimeter^®^ tool revealed that the three most frequently used words by students to describe a safe and inclusive healthcare environment for trans people were tolerance, respect, and empathy (Figure 4).

## 4. Discussion

According to the scientific literature, simulation through virtual resources is a very powerful training methodology that is particularly useful in the acquisition of non-technical skills [33].

Currently, nursing degree curricula are facing serious problems, such as a shortage of teaching staff to meet the high demand for university graduates in nursing [34]. There is also an urgent need to gradually incorporate new teaching strategies, such as those involving ICTs, which are geared towards meaningful learning [35]. To this end, teaching staff must understand how much and in what way ICTs can help in the teaching–learning process [36], as they play a mediating role between students and the technological resources that supplement the learning process [16].

Although some studies claim that clinical simulation is replacing real-life practice [37], there are numerous barriers to the implementation of clinical simulation, including a lack of access, lack of familiarity with the technology, and failure to share resources [19,38], or because of costs and technical difficulties, such as poor graphics [21]. In relation to the latter, some researchers argue that the use of ICTs is a good alternative to traditional teaching, contributing, to a great extent, to breaking down barriers [16].

Interesting tools to be implemented in university health science environments are virtual resources in either VLO or VLE formats, which have proven to be very effective in the teaching–learning process [20]. These methodologies encourage students to learn in a self-regulated, self-directed [20], critical, and flexible [16] manner, and improve their performance, motivation, and understanding of content [39].

Accordingly, the main practical implications offered by the use of these digital tools are improved problem solving and conflict resolution [40,41], improved applicability of theoretical knowledge [42], and clinical decision-making skills [5,30]. The improvement of procedural and communicative skills [9] for conducting personal [11] and clinical interviews [7] and understanding and empathising with the difficulties or barriers that people can face in health services are also important [6]. However, there is little research on student training using these technologies, especially to assess communication skills [13] and the development of clinical decision making [12].

Most of the identified studies, whose results are based on the pilot testing of virtual resources, fall within the field of physiotherapy [17,43,44,45,46]. Similar studies have also been found in the fields of physics [40], pedagogy [41], medicine [47,48,49], and dentistry [24,50]. We were only able to identify four Spanish studies [13,33,41,51], of which three were applied to nursing: one focused on increasing competence in a pre-anaesthesia assessment [33], one was dedicated to surgical training [51], and one was aimed at improving motivational interviewing skills [13]. Other studies applying VR and SVs to nursing focused on improving decision-making skills [52], developing critical thinking [53], assessing communication skills [54], and delivering training in the correct administration of medication [39]. Two studies are currently being pilot tested: one involving the use of VR and 360° videos centred around the development of clinical reasoning skills [55] and one aiming to improve emotional coping skills to reduce anxiety and increase self-confidence among students [56].

Our results are consistent with those of similar studies, which report statistical significance in terms of an increased levels of knowledge [11,42,45,48] and a high degree of satisfaction with the methodology used [11,13,17,33,41,45,51]. As shown in our study, the ability of these resources to foster empathy seems evident, echoing the findings of similar studies [24,42,50].

We were unable to find any studies assessing the impact of these teaching methodologies on improving the knowledge of gender diversity. However, although we found high statistical power and effect size values, our results should be interpreted with caution, as this was a quasi-experimental design with no control group, purposive sampling, and a relatively small sample size.

## 5. Conclusions

Firstly, we believe that universities must adjust their educational curricula to reflect the current legislation and to ensure that they meet the health needs of the population. In this sense, in Spain the National Agency for Quality Assessment and Accreditation should review the curriculum of the nursing degree and incorporate training and research in gender and sexual diversity in accordance with the provisions of Law 02/2021, of 7 June, of social equality and non-discrimination on the grounds of gender identity, gender expression, and sexual characteristics.

Nursing lecturers must be prepared to implement new teaching methodologies of proven utility in improving students’ academic success and performance. Secondly, this research sought to measure the impacts of the implementation of active methodologies, such as SVs and VR, on knowledge enhancement and skills acquisition. As shown, these approaches are becoming firmly established as learning activities that are highly appreciated and widely accepted. Thirdly, they have proven to be helpful in developing tolerance, respect, and empathy among students with trans patients or users in healthcare settings.

The results suggest that VLOs can be particularly effective in enhancing the learning of practical skills in a safe and controlled environment that builds confidence and helps them acquire the necessary skills to put them into practice. These results should be interpreted with caution as there was no control group and the sample size was relatively small.

The fact that no significance was found in questions 6, 7, and 9 (attitudinal skills block) suggests that the questions should be rephrased or emphasised during the initial short lesson.

A strength of our research was that no other studies assessed the use virtual resources for training university students in sexual and gender diversity in any discipline of the health sciences; more studies are needed on vulnerable populations and those at risk of exclusion, such as transgender people.

Finally, we believe that this study will help future research to obtain more accurate measurements when piloting virtual resources.

## Figures and Tables

**Table 1:** Descriptive statistics.

	N	Range	Mean	Std. Deviation	Variance
Pre-test	56	9	7.98	1.949	3.800
Post-test	54	10	13.22	2.034	4.138
Valid N (listwise)	54				

**Table 2:** Test statistics.

Question Pre- and Post-Tests	1	2	3	4	5	6	7	8	9	10		11	12	13	14	15
N	54	54	54	54	54	54	54	54	54	54	54		54	54	54	53
Chi-squared test ^b^	36.02		15.750		28.195			31.030			25.714				24.038	
Asymp. Sig.	<0.001		<0.001		<0.001			<0.001			<0.001				<0.001	
Exact Sig. (two-tailed)		<0.001 ^c^		0.049 ^c^		0.063 ^c^	1.000 ^c^		0.839 ^c^	<0.001 ^c^			<0.001 ^c^	<0.001 ^c^		<0.001 ^c^

^b^ Continuity-corrected. ^c^ Binomial distribution used.

## Data Availability

The data presented in this study are available on request from the corresponding author.

## Public Involvement Statement

There was no public involvement in any aspect of this research.

## Guidelines and Standards Statement

This manuscript was drafted against the Transparent Reporting of Evaluations with Nonrandomised Designs (TREND) [57] for the reporting of intervention evaluation studies using nonrandomised designs (quasi-experimental research design).

## Appendix A

1. Please select the correct statement regarding the pathologisation of transgenderism:
  - Transgenderism is no longer considered a psychiatric condition.
  - Both the WHO in its ICD and the APA in its DSM have removed transgenderism from their lists of psychiatric conditions.
  - Only the APA in its current DSM recognises transgenderism as a psychiatric condition.
  - None of the above is true.
2. The difference between a transsexual person and a transgender person is that:
  - The former undergoes treatments and/or surgeries that adjust their physical characteristics to the gender they identify with, while the latter does not necessarily accept their corporeality.
  - The former does not undergo treatments and/or surgeries that adjust their physical characteristics to the gender they identify with, while the latter does, as they do not accept their corporeality.
  - Both terms are used interchangeably.
  - Neither is correct.
3. Please select the correct statement:
  - Gender expression is an indication of a person’s sexual orientation.
  - Biological sex is the sex we are born with and is binary.
  - Gender identity is one’s sense of belonging to one gender or another and may or may not be in accordance with biological sex.
  - All of the above are correct.
4. Please select the main barrier to accessing healthcare for transgender people:
  - Prejudice.
  - Transphobia.
  - Ignorance.
  - All of the above.
5. Please select the correct statement:
  - There is no protocol for action in the Canary Islands, but efforts are being made to address the needs of the population.
  - The Canary Islands already have a protocol for the care of trans people that does not include intersex people.
  - The Canary Islands have an approved protocol for trans and intersex people.
  - None of the above is correct.
6. Please select the most appropriate option if you worked at a primary care facility and had a trans woman among your users:
  - I would always use the name appearing in her medical record.
  - If we do not know the person, we can use their surname and then ask for their first name and pronouns.
  - Until such time as this person’s registry office documents are amended, we must adhere to the patient record for legal reasons.
  - If possible, I would rather not have any transgender person in my quota.
7. When you meet a trans person for the first time, you:
  - Ask for their meaningful name and preferred pronouns.
  - Flatter them by telling them that they do not show it or that they hide it very well, as this empowers them and helps to create a therapeutic environment.
  - Ask for their deadname.
  - All of the above are correct.
8. Meeting the specific needs of the LGBTIQ+ population is included in:
  - The code of ethics for the nursing profession.
  - The Canary Islands protocol for the care of trans* people.
  - The Spanish Trans Law.
  - The 2030 Agenda.
9. Which law states that trans people have the right to be cared for by trained health professionals who are knowledgeable about the specific trans health reality?
  - Canarian Law 2/2021 of 7 June, on social equality and non-discrimination on grounds of gender identity, gender expression, and sexual characteristics.
  - Spanish Law 4/2023 of 28 February, for the real and effective equality of trans people and for the guarantee of the rights of LGTBI people.
  - Spanish Law 3/2007 of 15 March, regulating the registry rectification of the mention regarding the sex of persons.
  - None of the above. It is a duty of the health professional, not a right of the user.
10. Regarding the gynaecological/urological care of a trans man, we should NOT:
  - Include him in breast cancer screenings.
  - Include him in cervical cancer screenings.
  - Include him in prostate cancer screenings.
  - Provide him with pregnancy support and perinatal care.
11. What makes an environment safe for a transgender person?
  - Above all, the use of neutral and inclusive language.
  - A respectful approach.
  - The use of brochures, banners, and infographics in the facility.
  - All of the above are correct.
12. In your practice, you see a 4–5-year-old child whose parents are concerned about his or her behaviour and statements, which suggest that he or she is a transgender child. You:
  - Tell their parents that this is just another stage of sexual development, cases like this are very common, and not to make a big deal out of it, but to follow it up.
  - Tell their parents not to encourage this behaviour so that it is not perpetuated over time, they should be patient, and that their child will be normal in the future.
  - Actively listen to the child’s story, without conditioning him/her.
  - Refer the child to the paediatric endocrinologist to begin hormone treatment and avoid the development of secondary sexual characteristics that may generate dysphoria in the future.
13. Please select the most appropriate steps for trans children:
  - From the ages of 4 to 10 years: social transition; from the ages of 11 to 14 years: hormone treatment; ≥14 years old: puberty blockers.
  - From the ages of 4 to 10 years: puberty blockers; from the ages of 11 to 14 years: social transition; ≥14 years old: hormone treatment.
  - From the ages of 4 to 10 years: social transition; from the ages of 11 to 14 years: puberty blockers; ≥14 years old: hormone treatment.
  - None of them is correct. Trans children cannot receive any type of hormone treatment until they come of age (at 18 years old in the Spanish context).
14. What would be the most appropriate way to address the sexual health of transgender people?
  - By asking them openly if and how they have sexual relations.
  - By asking them ‘Do you have any questions about your sexual health?’
  - By asking them ‘Do you mind if I ask you a few questions about your sexuality?’
  - The sexual sphere of transgender people should not be assessed by nurses, as they may feel intimidated.
15. You work at a hospital. A trans man is being admitted to your unit for a mastectomy and you are told: ‘His name is Anthony, but his medical records and ID card say his name is Patricia... I need you to give me a bed for his admission.’ Please select the correct option:
  - If his name does not match his legal documents and medical records, he cannot be admitted.
  - He will be admitted to a ward with other females in accordance with his sex at birth.
  - If possible, he/she will be given a bed in a private room and admitted alone.
  - He should be admitted to a ward with other males in accordance with his gender.
